# Exophytic growth of a neglected giant subcutaneous Leiomyosarcoma of the lower extremity. A case report

**DOI:** 10.1186/1477-7800-5-11

**Published:** 2008-05-21

**Authors:** Marina Angeloni, Francesco Muratori, Nicola Magarelli, Byron E Chalidis, Riccardo Ricci, Barbara Rossi, Giulio Maccauro

**Affiliations:** 1 Orthopaedic Department, Catholic University of Rome, Italy; 2 Radiological Department, Catholic University of Rome, Italy; 3 Department of Orthopaedic Surgery, University of California, San Francisco, CA, USA; 4 Department of Pathology, Catholic University of Rome, Italy

## Abstract

**Background:**

Superficial leiomyosarcoma is an exceedingly uncommon malignant tumor which could be located either to cutaneous or subcutaneous tissues. Increased mass size and depth, advanced tumor staging and inadequate surgical excision are the main prognostic factors for poor result.

**Case presentation:**

We report a rare case of a 71-year-old man with an extensive exophytic lesion (12 × 10 cm) in the anterior-medial side of the proximal right tibia. The lesion was painless and consistently neglected by the patient until a skin trauma caused ulceration of the affected area. Magnetic Resonance Imaging revealed a soft-tissue mass which was well defined from the surrounding bone and muscles. As initial biopsy in another hospital hadn't clarified the true nature of the lesion, new samples were taken and the diagnosis of leiomyosarcoma was established. Laboratory examination showed no distant metastasis and wide excision of the neoplasm was decided. After tumor resection, the remaining soft tissue and skin defect was covered with a gastrocnemius myocutaneous flap. The postoperative period was uneventful and wound healing was followed by local radiotherapy and systemic chemotherapy. At 3 years follow up, no recurrence or metastasis was identified and the patient was able to walk and stand without impairment of his ambulatory status.

**Conclusion:**

Proper surgical management of soft tissue leiomyosarcoma continues to remain the cornerstone of treatment efficacy and the most important prognostic factor for patients' survival. Reconstruction of the remaining soft tissue defect should be always performed at the same operative time when removal of giant size tumors leaves an uncovered cavity with an inadequate sleeve of muscular and skin tissues.

## Background

Superficial Leiomyosarcoma (LMS) is a quite uncommon tumor which is mainly developed in the proximal portion of the lower limbs [1-3]. The lesion can be appeared as a single nodular mass or multiple swelling tumours [2,4]. Although it may occur in any age, superficial LMS is more prevalent between the fifth and seventh decade of life. Furthermore, a male predominance with a male-to-female ratio of 2:1 was also described [1,2]. The tumor could be located either cutaneously or subcutaneously. In the first case, it is arisen from the limb extensor surfaces where a greater density of hair structures (arrectores pilorum muscles) exists [1,2,5]. It is frequently quite small-less 2 cm-causing local changes like skin discoloration, umblication and ulceration [1,2].

Subcutaneous soft tissue Leiomyosarcomas (LMSs) are apparently larger and vascular in their origin, grow faster and they may produce similar changes in the skin. Their incidence is even rarer accounting for 2.3 – 5.3% of all malignant soft tissue tumors [1,5] and 4 – 6,5% of all soft tissue sarcomas [6,7]. As clinical signs are not strongly suggestive of malignancy and vary from person to person, the definitive diagnosis may be delayed until the later stage of the disease [2]. Wide excision constitutes the current treatment of choice [1,2,5,8-15] while radiotherapy (RTP) and chemotherapy (CTP) may be considered in high grade lesions [1,2,10,12-15].

We report a rare case of a giant subcutaneous LMS of the proximal tibia. Wide excision of the tumour in combination with local RTP and systemic CTP led to good functional outcome and no recurrence until the latest follow up (3 yrs postoperatively).

## Case presentation

**Figure 1:**
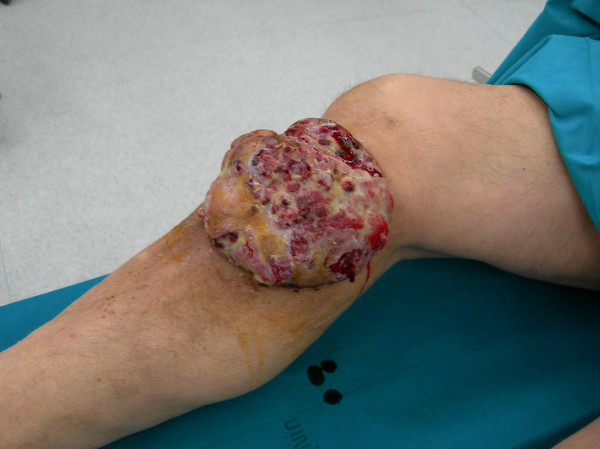
Macroscopic appearance of the exophytic mass in the anterior-medial side of the right leg. The mass showed nodular growth pattern and the cut surfaces were ulcerated and bloody with irregular areas of necrosis.

**Figure 2:**
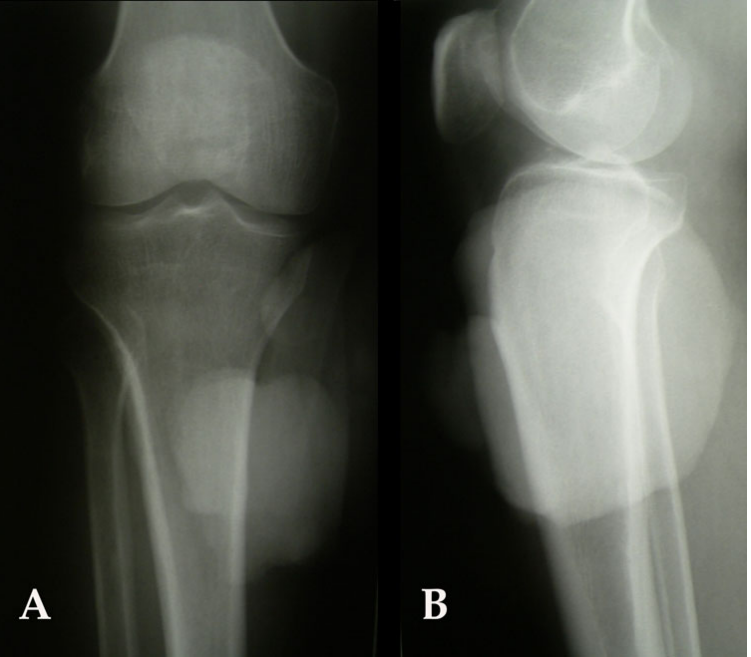
Anteroposterior **(A) **and Lateral **(B) **X-Ray view of the right knee. A soft tissue mass in the anterior-medial side of the proximal tibia with no evident continuity with the bone or cortical invasion was apparent.

**Figure 3:**
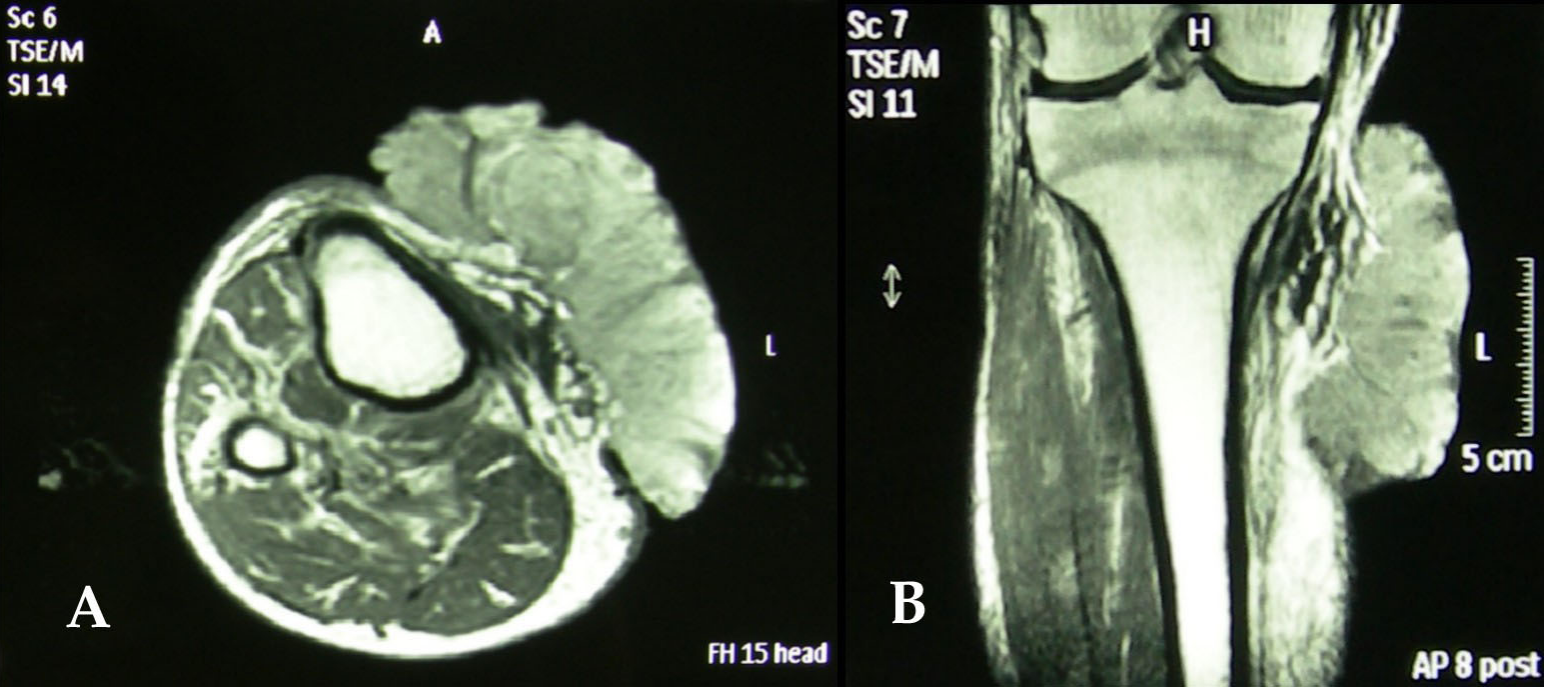
Axial **(A) **and Coronal **(B) **MRI T1-weighted image of the right proximal tibia. A large homogeneous mass which was invaded the adjacent soft tissue and extended along the medial surface of cutaneous plane was visible. No signs of bone tissue infiltration, or abnormal intensity in muscle tissues could be seen.

A 71-year-old man, without medical history, referred to Tumor Outpatient Clinic of the Hospital due to an extensive exophytic mass of the anterior-medial side of the right proximal tibia. The lesion was appeared ulcerated and bloody with irregular areas of necrosis and haemorrhage and its size was 12 × 10 cm with collar of around 10 cm. (Figure 1). The patient reported that the mass was completely asymptomatic and its growth was slow. He wasn't worried about its nature until an innocuous trauma caused ulceration at tumor site 2 weeks ago. Plane X-Rays showed a radiolucent lesion adjacent to the anterior-medial side of the proximal tibia without cortical disruption or continuity with the bone (Figure 2). Magnetic Resonance Imaging (MRI) confirmed the suprafascial character and extension of the lesion and revealed no clear relationship with the surrounding muscles and bone (Figure 3). The previously performed open biopsy couldn't clarify the true nature of the lesion as the described diagnosis was "unspecified soft tissue sarcoma". According to the above findings and after patient's referral, new samples were received and histologic examination showed a soft tissue leiomyosarcoma. Subsequent Computed Tomography (CT) scans of chest and abdomen and total bone scan excluded the presence of systemic lesions or metastasis and the patient was scheduled to undergo surgical resection of the lesion with additional removal of a sleeve of surrounding tissues.

**Figure 4:**
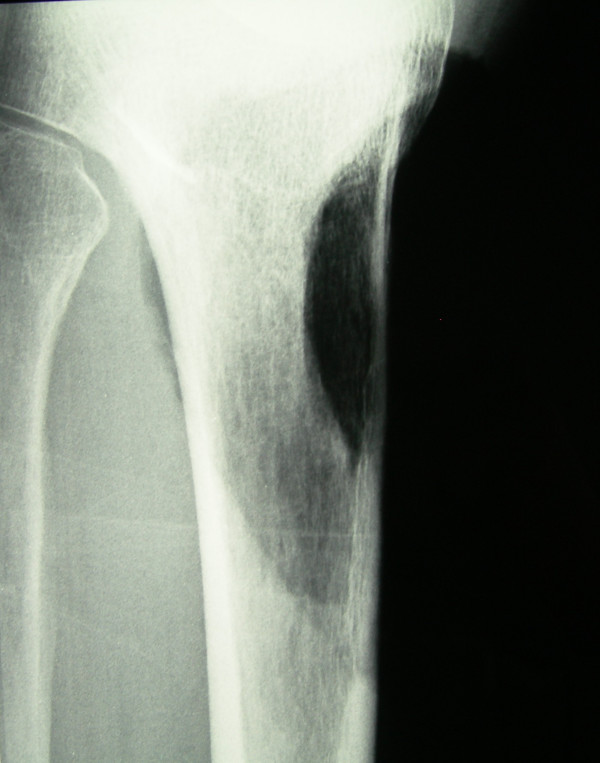
Post-operative Anteroposterior X-Ray view of the proximal tibia. Bone resection was decided for the achievement of wide and tumor-free surgical margins.

**Figure 5:**
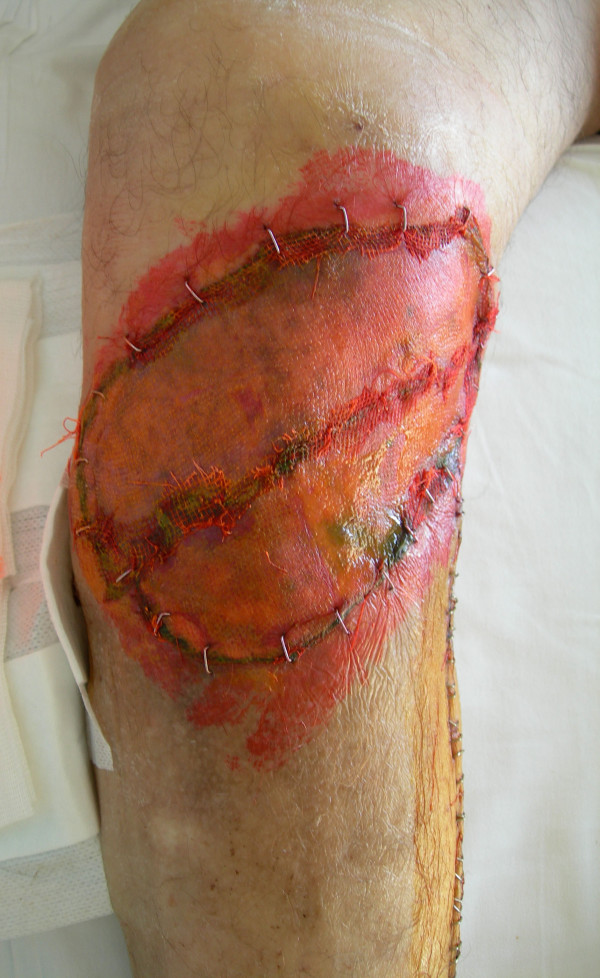
Macroscopical post-operative aspect of patient's leg. Adequate coverage of defects of the tibia was obtained with the gastrocnemius myocutaneous flap.

Under general anaesthesia and tourniquet application, *en block *wide excision of the mass was performed. As the tumor was very close to the bone, a portion of the anterior-medial cortex of the proximal metaphysis of the tibia was also removed to facilitate at least 2 cm resection margins circumferentially (Figure 4). After tumor excision, a large skin and soft tissue defect was remained and transplantation of gastrocnemius myocutaneous flap was deemed necessary for covering the defect (Figure 5). At the end of the procedure, the tourniquet was released and the vascular integrity of the flap and limb was evaluated.

**Figure 6:**
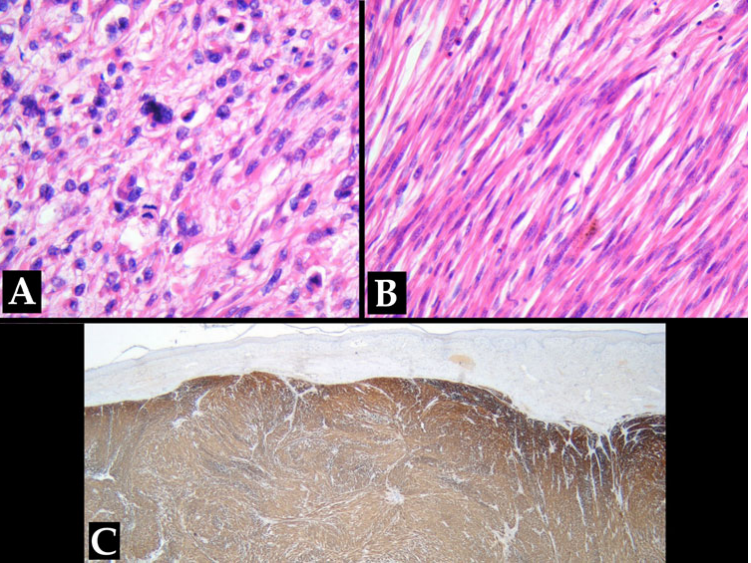
Microscopic features at high magnification included **(A) **the high number of pleomorphic cells with frequent mitosis and **(B) **the focally fused cells with central nucleus (hematoxylin and eosin, × 400). **(C) **Immunohistochemical staining Caldesmon. According to this technique a diagnosis of grade III LMS was made (caldesmon, × 20).

At histological analysis, multiple elongated cells of mesenchymal origin surrounded by myofibrils and many polygonal epithelioid cells with evident pleiomorphism and elevated mitotic index were found. The surgical margins were free of disease and some necrotic areas were recognised as well. The positive immunohistochemical staining for smooth muscular actin, vimentin and caldesmon led to diagnosis of grade III LMS according to the grading scale modified from Broders et al. and Angervall et al. [16,17] (Figure 6).

The postoperative course was uneventful and no local or systemic complications were encountered. Due to the weakening of the proximal tibia from the excision of the anterior-medial cortex, partial weight bearing for 6 weeks was advised. After wound healing the patient was submitted to the Oncology Department for local RTP and systemic CTP.

At the latest follow up visit (3 years) clinical and laboratory examination (X-Rays, MRI, Bone Scan) didn't reveal any signs of tumor recurrence or metastasis. The patient could stand and walk without any restriction and he was able to perform his usual daily activities.

## Discussion

The distinction between cutaneous and subcutaneous LMS is of primary importance for prognostic reasons [2,8,10]. Although the cutaneous LMSs are associated with local recurrence in about 30–50% of cases only a low incidence of 0–10% of metastasis has been described [1,2,15,18]. On the other hand, subcutaneous LMSs may develop local recurrence in about 40–60% of patients and distant metastasis in 20 to 60% of patients [1,2,5,8,10,12,15]. As a result, the mortality may be raised up to 30–40% [2,18]. Despite the fact that tumor location and depth affect the recurrence rate and metastatic risk, the mitotic activity seems not to have a similar effect [1,2,8].

In the current patient, the size of the tumor (12 × 10 cm) was higher than the previously reported cases. Specifically, the mean size reported in literature for superficial LMSs is about 5 cm, with a wide range from 0.5 to 19 cm [2,4,8,19]. Some authors described cases of LMSs with larger size (up to 35 cm), but in their series they didn't distinguish the superficial soft tissue LMSs from non-visceral deep LMSs [15,20]. Large tumors (>5 cm) are considered to have worst prognosis in terms of recurrence and metastasis [1,8,12-15]. In a multivariate analysis of 267 patients with leiomyosarcoma, Miyajima et al. found that tumour size and advanced staging were the only factors that were correlated independently with decreased survival [20].

Wide excision of the lesion is of outmost importance as it is strongly correlated with disease-free survival [1,2,8,10,12-15]. Massi et al. in a retrospective study of 62 patients with extremity soft tissue leiomyosarcoma reported that seven out of the nine patients who had developed one or more local recurrences had been surgically treated with either marginal or intralesional excision [13]. Similarly, Svarvar et al. in a large study of 225 patients with leiomyosarcoma which was based on Scandinavian Sarcoma Group Register showed that the inadequate local treatment was the main risk factor for local recurrence [15].

Many authors have been supported the application of postoperative RTP and CTP in high grade LMSs [1,2,10,12-15]. However, the role of adjuvant therapy in LMSs is still controversial as no sufficient statistical evidence exists regarding their efficacy. The above treatment modalities may have a higher impact on local and systemic tumor respond when adequate excision of large and high grade lesions could not be achieved [13-15].

## Conclusion

Even though delayed diagnosis and giant size of soft tissue leioymyosarcomas may adversely affect the final result, successful recovery could be anticipated. Special focus should be given to careful and meticulous wide excision of the mass as the role of chemotherapy and radiotherapy is still blur and unknown.

## Consent

Written informed consent was obtained from the patient for publication of this case report and any accompanying images. A copy of the written consent is available for review by the Editor-in-Chief of this journal

## Competing interests

The authors declare that they have no competing interests.

## Authors' contributions

GM, the chief surgeon in charge of the case prepared, participated in the design of the paper and in the drafting of the manuscript. MA, FM, BR participated equally in the design of the paper and in the drafting of the manuscript. RR participated in the study of macroscopic and microscopic features of the lesion. NM participated in the study of the radiological data. BEC revised critically the final version of the manuscript. All authors read and approved the final manuscript.
